# Efficacy of Mineral Trioxide Aggregate (MTA) as a Reparative Material in Iatrogenic Furcal Perforations in Mandibular Molars

**DOI:** 10.7759/cureus.53206

**Published:** 2024-01-29

**Authors:** Ayesha Nazeer, Amara Nazir, Syeda A Manzoor, Muhammad A Khan, Zunaira Shaukat, Mehvish Saleem, Mustafa Sajid, Muhammad Kashif

**Affiliations:** 1 Operative Dentistry, Nishtar Institute of Dentistry, Multan, PAK; 2 Operative Dentistry, Bakhtawar Amin Medical and Dental College, Multan, PAK; 3 Oral Medicine, Bakhtawar Amin Medical and Dental College, Multan, PAK; 4 Dentistry, Multan Medical and Dental College, Multan, PAK; 5 Dentistry, Bakhtawar Amin Medical and Dental College, Multan, PAK; 6 Oral Pathology, Bakhtawar Amin Medical and Dental College, Multan, PAK

**Keywords:** periapical lesion, efficacy, mta, mineral trioxide aggregate, furcal perforations

## Abstract

Background/Objectives: Mineral trioxide aggregate (MTA) is widely recognized as one of the most biocompatible materials for perforation repairs during root canal treatment (RCT). Experimental evidence has consistently demonstrated MTA's superior sealing ability and biocompatibility compared to various dental materials, including amalgam, intermediate restorative material, zinc oxide eugenol cement, and resin-modified glass ionomer cement. This study aimed to assess the efficacy of MTA as a reparative material in iatrogenic furcal perforations during RCT.

Materials & methods: A descriptive cross-sectional study was conducted from May 18, 2021, to November 17, 2021, at the Department of Operative Dentistry, Nishtar Institute of Dentistry, Multan, Pakistan. Seventy-six patients aged 18-60 years, of both genders, who developed iatrogenic furcal perforations during procedures were included. Patients with fractures or endo-perio lesions identified during clinical and radiographic examinations were excluded. Isolation was achieved using a rubber dam. The perforation site was cleaned and irrigated with 1% sodium hypochlorite to control hemorrhage and enhance visualization. Following the manufacturer's recommendations, the perforation site was sealed with MTA mixed with sterile saline.

Results: The age range in this study was 18 to 60 years, with a mean age of 42.09 ± 9.69 years. Most patients (56.78%) were between 41-60 years old. Out of the 76 patients, 46 (60.53%) were male, and 30 (39.47%) were female, resulting in a male-to-female ratio of 1.5:1. The study found that MTA's efficacy as a reparative material in iatrogenic furcal perforations was observed in 61 (80.26%) patients. A 6-month follow-up revealed no periodontal ligament breakdown, demonstrating the efficacy of MTA as a reparative material in iatrogenic furcal perforations.

Conclusion: This study concludes that the efficacy of MTA as a reparative material in iatrogenic furcal perforations is remarkable and significant.

## Introduction

Iatrogenic furcal perforation is an unfavorable encountered complication occurring during endodontic treatment that may lead to its failure. Iatrogenic furcal perforation may lead to an inflammatory reaction in the periodontal ligament that should be managed. The prognosis of iatrogenic furcal perforation depends on the time of repair, size of perforation, pre-existing periodontal disease, and pulp vitality status of the involved tooth [1].

Furcal perforation stands out as one of the most undesirable and common mishaps encountered during endodontic practice. This type of accident often results from using burs with inappropriate dimensions or improper directions while removing the pulp chamber ceiling and identifying root canal orifices. Furcal and/or root perforation prognosis is unfavorable. Tooth extraction or perforation sealing using different materials, such as endodontic or restorative cement, is usually recommended and chosen based on prognosis. The best clinical results were obtained using calcium hydroxide with different clinical strategies [2].

However, large-sized furcal perforations do not respond favorably to calcium hydroxide, possibly due to its restricted physical and chemical properties. Thus, other materials have been proposed to solve this problem, such as calcium silicate-based cement, which has demonstrated excellent biological and clinical results. These cements have currently caused some scientific enthusiasm, including new chemical modifications and/or associations with different vehicles to improve their clinical behavior, handling, and biological properties. On the other hand, a long-term clinical assessment using this material is still unclear [3]. MTA is one of these calcium silicate cement that was introduced in the 1990s and extensively studied to be used for perforation repairs, apexification, regenerative procedures, apexogenesis, pulpotomies, and pulp capping [4].

MTA has been recognized as a highly effective material for various dental procedures, including perforation repair, pulp capping, and apexification [2]. Comprising various mineral oxides, MTA is a calcium silicate-based cement with a pH of 12.5. Its key components include tricalcium silicate, tricalcium aluminate, and tricalcium oxide. In the presence of moisture, MTA typically sets in about four hours [3].

Different studies conducted in the past showed that MTA is nontoxic, nonabsorbable, radiopaque, and bactericidal. So, MTA is considered one of the most biocompatible materials for perforation repairs [4,5]. MTA demonstrated superior sealing capacity and biocompatibility in experimental settings compared to various previously employed dental materials, including amalgam, intermediate restorative material, zinc oxide eugenol, and resin-modified glass ionomer cement [6].

Furthermore, MTA offers effective sealing against microleakage resulting from perforations. Its biocompatible nature poses MTA as an ideal material to promote or improve periodontal attachment regeneration, promoting osteogenesis and aiding cementum formation [7]. In a study carried out at the University of Bangladesh, twenty cases of iatrogenic furcal perforation were treated by sealing with MTA, followed by endodontic and coronal restoration treatment. The findings revealed a successful sealing outcome in 19 out of 20 teeth, yielding an impressive success rate of 95% for MTA [8]. In another study conducted at the University of Florence, MTA was found to be successful in 9 out of 10 cases, i.e., 90% [9]. Another study of 128 cases older than 18 years of age showed a success rate of 73.3% in patients with iatrogenic furcal perforations treatment [10]. Also, in a study conducted in 2017, patients showed complete recovery with a success rate of 95.6% with MTA [11].

This study aimed to evaluate the efficiency of MTA as a reparative material in iatrogenic furcal perforation sealing. Literature shows that MTA is a highly successful material in repairing iatrogenic furcal perforation, but different results have been reported in the literature. Moreover, limited data is available regarding the iatrogenic furcal perforation repair success rate with MTA, and no local data is available. Hence, we conducted this study to find local evidence on the use of MTA for iatrogenic furcal perforation sealing to improve the outcome and success rates in the treatment of these cases.

## Materials and methods

This descriptive cross-sectional study was undertaken in the Department of Operative Dentistry at Nishtar Institute of Dentistry, Multan, spanning from May 18, 2021, to November 17, 2021. The Ethical Review Committee of Nishtar Institute Multan granted approval to perform this study (CPSP/REU/DSG-2017-102-2123). The sample size of 76 cases was determined based on a calculated success rate of 73.3% for MTA in repairing iatrogenic furcal perforations, considering a confidence level of 95% and a margin of error of 10.0%.

The sampling technique employed in this study was non-probability, specifically consecutive sampling. Participants aged between 18 and 60 years, of both genders, undergoing root canal treatment (RCT) due to irreversible pulpitis, and subsequently experiencing iatrogenic furcal perforation during the procedure were included. Teeth with fractures, as assessed during clinical and radiographic examinations, and those with endo-perio lesions (indicating breakdown in both endodontic and periodontal tissues) were excluded. Following approval from the hospital's ethical review committee, patients presenting at the Outpatient Department (OPD) of the Department of Endodontics, Nishtar Institute of Dentistry, Multan, were selected based on the inclusion criteria. Informed written consent was obtained from each participant.

Baseline characteristics, including age, gender, smoking status, diabetes mellitus (DM), and betel nut chewing habits, were documented.

**Figure 1:**
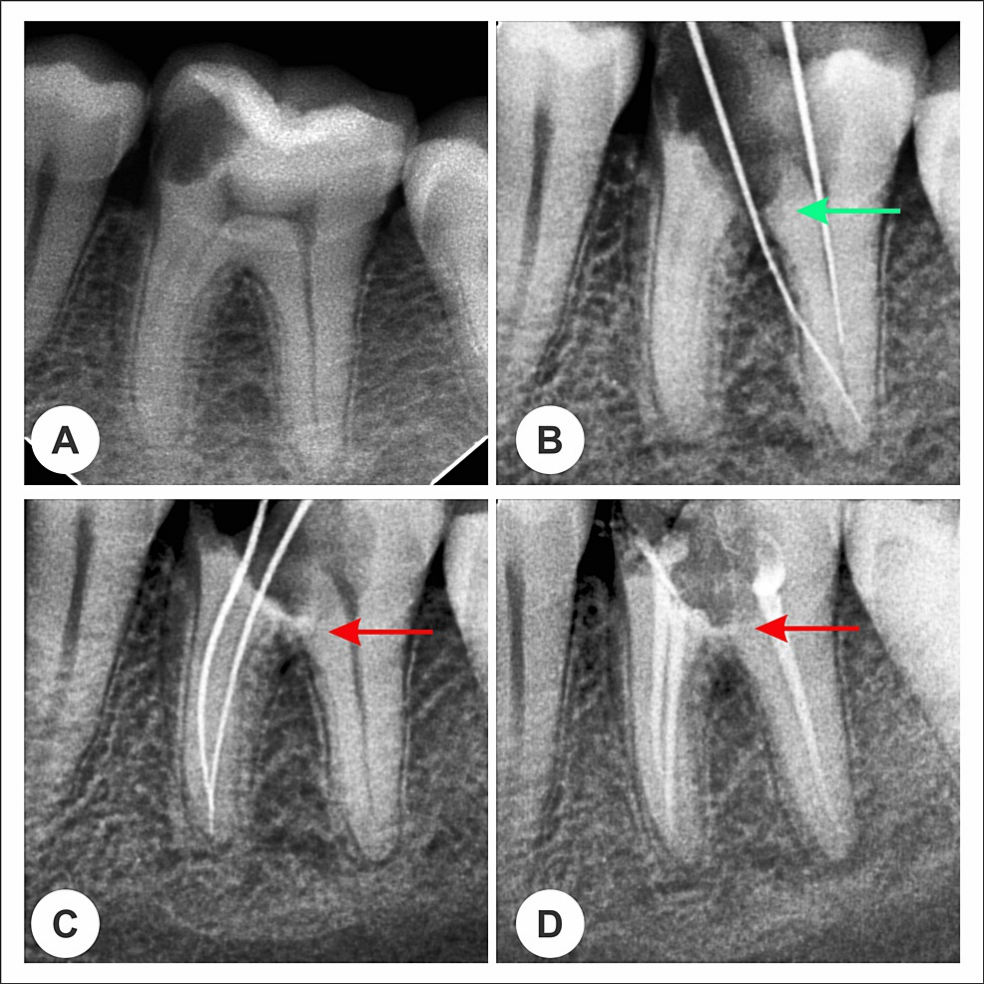
Radiographic presentation of furcal perforation and its repair with MTA, (A) Class II cavity/carious lesion involving pulp and evidence of periapical changes, (B) Green arrow shows furcal perforation during access cavity preparation, (C) Red arrow shows the repair/sealing of furcal perforation with MTA, (D) Repaired/sealed perforation and finally obturated mandibular 1st molar tooth

Subsequently, a rubber dam was employed for isolation. The perforation site underwent cleaning and irrigation with 1% sodium hypochlorite to manage hemorrhage and facilitate site visualization. Following the manufacturer's recommendations, the perforation site was then sealed with MTA mixed with sterile saline. MTA was covered with a cotton pellet moistened with distilled water, and the cavity was temporarily restored using intermediate restorative material (IRM). Root canal treatment was completed the following day, and the tooth was restored. A radiograph taken at the time of treatment revealed radiopaque features, with no loss of tooth structure at the site of iatrogenic furcal perforation, confirming successful sealing (Figure 1). A 6-month follow-up showed no breakdown of the periodontal ligament, indicating the efficiency of MTA as a reparative material in iatrogenic furcal perforation (Figure 1).

All this data was recorded on a proforma and subsequently entered and analyzed using SPSS version 20.0 (IBM, NY, USA). The variable "age" was presented as mean ± SD, while variables such as gender, smoking status, betel nut chewers, diabetics, and efficacy were presented in terms of frequency and percentages. Data were stratified to control for potential effect modifiers (including age, gender, smoking status, betel nut chewers, and diabetics). The chi-square test was employed to compare success rates in the various stratified groups following stratification. A p-value of ≤0.05 was considered statistically significant.

## Results

The age range in this study spanned from 18 to 60 years, with a mean age of 42.09 ± 9.69 years. The majority of participants, comprising 43 individuals (56.78%), fell within the 41 to 60 years age bracket. Out of the total 76 patients, 46 (60.53%) were male, while 30 (39.47%) were female, resulting in a male-to-female ratio of 1.5:1. The distribution of patients based on diabetes mellitus, smoking habits, and betel nut chewing is illustrated in Table 1.

**Table 1:** Baseline characteristics of study subjects with iatrogenic furcal perforation

Variables	Value/Number/percentage
Mean age (years)	42.09 ± 9.69
Age groups	
18-40	33 (43.22%)
41-60	43 (56.78%)
Gender	
Male	46 (60.53%)
Female	30 (39.47%)
Diabetic status	
Yes	12 (15.79%)
No	64 (84.21%)
Smoking status	
Yes	16 (21.09%)
No	60 (78.91%)
Betel nut chewing	
Yes	13 (17.11%)
No	63 (82.89%)

**Figure 2:**
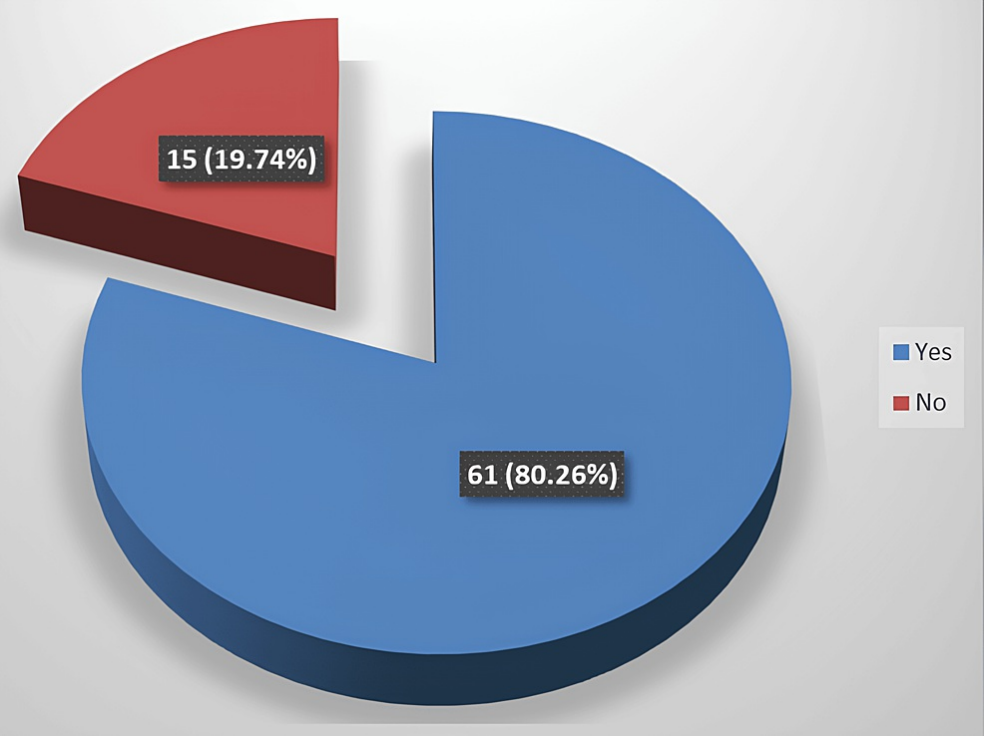
Efficacy of MTA as a reparative material in iatrogenic furcal perforations

In our investigation, the efficiency of MTA as a reparative material in iatrogenic furcal perforations was observed in 61 patients, constituting 80.26% of the study cohort (Figure 2).

Table 2 shows the cross-tabulation of the efficiency of MTA in addressing furcal perforation with other study variables. The variables considered include age groups, gender, diabetes mellitus, smoking, and betel nut chewing. For each variable, the table provides counts and percentages for individuals responding affirmatively ("Yes") or negatively ("No") to MTA treatment, accompanied by p values indicating statistical significance. Notable observations include a higher efficacy in the 18-40 age group (81.82%) compared to 41-60 (79.07%), a slightly higher efficacy in females (83.33%) than males (78.26%), and no significant difference in efficacy based on diabetes status. Smoking showed a trend towards significance, with 62.50% efficacy in smokers and 85.0% in non-smokers. Similarly, betel nut chewing exhibited no substantial difference in MTA efficacy. Overall, these findings contribute to understanding the nuanced relationships between patient characteristics and the effectiveness of MTA in treating furcal perforation.

**Table 2:** Comparison of clinical variables with efficacy of MTA in iatrogenic furcal perforation

Variables	Efficacy of MTA in furcal perforation		*p* value
	Yes	No	
Age groups			0.765
18-40	27 (81.82%)	06 (18.18%)	
41-60	34 (79.07%)	09 (20.93%)	
Gender			0.587
Male	36 (78.26%)	10 (21.74%)	
Female	25 (83.33%)	05 (16.67%)	
Diabetes Mellitus			1.000
Yes	10 (83.33%)	02 (16.67%)	
No	51 (79.69%)	13 (20.31%)	
Smoking			0.073
Yes	10 (62.50%)	06 (37.50%)	
No	51 (85.0%)	09 (15.0%)	
Betel nut chewing			0.713
Yes	10 (76.92%)	03 (23.08%)	
No	51 (80.95%)	12 (19.05)	

## Discussion

In the past, various materials have been employed for sealing furcal perforations, such as amalgam, IRM, SuperEBA, Cavit, Glass Ionomer, and composites. However, none of these materials meet the criteria of an ideal repairing material, which encompasses the ability to effectively seal and biocompatibility [12]. Since its introduction in 1993, MTA has emerged as the preferred choice for perforation seals, retrograde filling, pulp capping, and apexification [13]. The fundamental compounds within MTA consist of several mineral oxides, dictating their chemical and physical characteristics [14]. MTA is a mineral powder consisting of hydrophilic particles, with tricalcium silicate, tricalcium aluminate, tricalcium oxide, and other mineral oxides as its primary components. With a pH of 12.5, comparable to calcium hydroxide, MTA sets in the presence of moisture in approximately 4 hours [14]. Presently available in gray (GMTA) and white (WMTA), WMTA contains lower levels of iron, aluminum, and magnesium compared to GMTA.

When employed as a reparative substance for furcal perforations, MTA possesses a multitude of advantageous characteristics, including proficient sealing capabilities, biocompatibility, bactericidal activity, radiopacity, and the capacity to solidify in the presence of blood [14,15]. Numerous studies have demonstrated superior outcomes in perforation repairs using MTA compared to repairs performed with materials such as amalgam, IRM, Zinc oxide Eugenol (ZOE), or SuperEBA, assessed through dye and bacteria leakage methods [14]. The biocompatibility of MTA renders it a fitting material for treating root perforations with the objective of regenerating a periodontal attachment [12,13]. Additionally, it has the capability to induce osteogenesis and cementogenesis [15,16]. Roots subjected to MTA treatment displayed an absence of inflammatory tissue layers, with the formation of root cementum that adhered to the MTA [15].

This study assessed the effectiveness of MTA as a reparative material for iatrogenic furcal perforations. The age range of participants in this investigation spanned from 18 to 60 years, with a mean age of 42.09 ± 9.69 years. The majority of patients, comprising 43 individuals (56.78%), fell within the age range of 41 to 60 years. Out of the 76 patients, 46 (60.53%) were male, and 30 (39.47%) were female, resulting in a male-to-female ratio of 1.5:1.

In our study, the efficacy of MTA as a reparative material in iatrogenic furcal perforations was evident in 61 patients, constituting 80.26% of the cases. A study conducted at the University of Bangladesh involving the sealing of twenty cases of iatrogenic furcal perforation with MTA, followed by endodontic and coronal restoration, reported a success rate of 95% in 19 out of 20 teeth [8]. Similarly, research at the University of Florence revealed a success rate of 90% in 9 out of 10 cases treated with MTA [9].

In a separate investigation involving 128 cases aged 18 years and older, a success rate of 73.3% was observed in patients with iatrogenic furcal perforation [10]. Another study demonstrated a complete recovery in patients, achieving a success rate of 95.6% with the use of MTA [11].

As indicated by Torabinejad et al., the observed minimal bacterial leakage when using MTA as a reparative material can be attributed to its effective sealing capability rather than any inherent antimicrobial properties of the substance [17]. Numerous studies have affirmed that MTA lacks a significant inhibitory effect on the growth of various oral bacteria [17,18]. In contrast, Eldeniz et al., in their assessment of leachable components from selected root-end filling materials, discovered that set samples of ProRoot MTA cement completely inhibited Pseudomonas aeruginosa and either delayed or restricted the growth of Enterococcus faecalis [19]. Consistent findings were reported in other studies investigating the antimicrobial characteristics of MTA [1,19].

MTA comprises SiO_2_, K_2_O, Al_2_O_3_, Na_2_O, Fe_2_O_3_, SO_3_, CaO, Bi_2_O_3_, MgO, CaO, KSO_4_, NaSO_4_ insoluble residues, and crystalline silica. It exhibits favorable biological compatibility, promoting alkaline phosphatase activity, the formation of mineralized nodules, and cell proliferation. Additionally, it demonstrates a lower occurrence of inflammatory chemical mediators, facilitating local tissue repair [18]. While it initiates an immediate inflammatory reaction, there is a decrease in the number of inflammatory cells after 60 days, accompanied by significant periodontal space repair under conditions similar to normal tissue [20].

In addition to the favorable outcomes observed in the investigation concerning the sealing effectiveness of MTA in furcal perforations, several limitations should be acknowledged. These include a relatively small sample size, a brief study duration, the absence of histopathological evidence for the perforation site, and the omission of cone beam tomography (CBCT) for a comprehensive dimensional assessment of the repaired/sealed perforation site.

## Conclusions

MTA was chosen as the preferred material due to its commendable biological properties. Radiographic and computed tomography analyses confirmed the regression of the periodontal space, aligning with previously documented microscopic observations. Consequently, MTA demonstrated a favorable clinical outcome as a sealing material for furcal perforations repair. The findings of this research affirm the high efficiency of MTA as a reparative material in iatrogenic furcal perforations repair. As a result, we advocate the routine use of MTA in iatrogenic furcal perforations repair to mitigate community morbidity. While various modifications in composition and/or handling techniques have been proposed to enhance MTA utilization, the present study adhered to the manufacturer's instructions, maintaining the original composition.
